# Supplemental Microalgal Iron Helps Replete Blood Hemoglobin in Moderately Anemic Mice Fed a Rice-Based Diet

**DOI:** 10.3390/nu12082239

**Published:** 2020-07-27

**Authors:** Rohil S. Bhatnagar, Dennis D. Miller, Olga I. Padilla-Zakour, Xin Gen Lei

**Affiliations:** 1 Department of Food Science, Cornell University, Ithaca, NY 14853, USA; rb664@cornell.edu (R.S.B.); ddm2@cornell.edu (D.D.M.); oip1@cornell.edu (O.I.P.-Z.); 2 Tata-Cornell Institute for Agriculture and Nutrition, Cornell University, Ithaca, NY 14853, USA; 3 Department of Animal Science, Cornell University, Ithaca, NY 14853, USA

**Keywords:** inulin, iron bioavailability, mice, microalgae, phytase, rice-based diet

## Abstract

Iron deficiency anemia affects 1.2 billion people globally. Our objectives were to determine if (1) supplemental iron extracted from defatted microalgae (*Nannochloropsis oceanica,* DGM) and (2) a combination of minute amount of plant phytase and inulin could help replete hemoglobin in anemic mice. Mice (7 weeks old) were fed a control diet (6 mg Fe/kg). After 10 weeks, the mice were assigned to three treatments: control, control + DGM iron (Fe-DGM, 39 mg Fe/kg), or control + 1% inulin + 250 units of phytase/kg (INU−PHY, 6 mg Fe/kg). The mice had free access to diets and water for 6 weeks. The Fe-DGM group had elevated blood hemoglobin (*p* < 0.01) and a two-fold greater (*p* < 0.0001) liver non-heme iron over the control. Strikingly, the INU-PHY group had 34% greater non-heme iron than the control, despite the same concentrations of iron in their diets. Fe-DGM group had altered (*p* < 0.05) mRNA levels of hepcidin, divalent metal transporter 1, transferrin and transferrin receptor 1. Iron extracted from defatted microalgae seemed to be effective in alleviating moderate anemia, and INU-PHY enhanced utilization of intrinsic iron present in the rice diet. Our findings may lead to a novel formulation of these ingredients to develop safer and bioavailable iron supplements for iron-deficient populations.

## 1. Introduction

Even though iron is the fourth most abundant element in the earth’s crust, iron deficiency anemia affects more than 1.2 billion people worldwide [1]. It has been estimated that iron deficiency, without anemia, is at least twice as frequent [2]. The burden of iron deficiency is most prevalent in resource-limited settings, due to insufficient iron intake and/or poor bioavailability through monotonous plant-based diets [3]. In such areas, fortification has been considered to be a successful and cost-effective remedy [4]. However, iron fortificants with relatively high bioavailability negatively affect the sensory profile of foods, limiting their palatability. In contrast, fortificants that do not cause these sensory issues often suffer from low bioavailability [5]. Additionally, some iron fortificants and iron-containing micronutrient powders may also cause adverse health effects in children, especially in malaria-endemic areas [6,7,8]. Thus, better iron sources that can be supplemented into food products to improve iron nutrition are needed.

Most non-heme iron in foods occurs as ferric ion (Fe^3+^). It is first reduced to ferrous ion (Fe^2+^) by duodenal cytochrome B (DCytB), located on the apical membrane of enterocytes [9]. The entry of iron into the cell is mediated by the apical duodenal metal transporter 1 (DMT1) [10]. Inside the cell, iron is either stored as ferritin or released into circulation via the basolateral iron exporter, ferroportin (FPN). Ferrous ion (Fe^2+^) from the FPN is first oxidized before being released into the blood stream to bind with transferrin (Tf). The iron homeostasis is orchestrated by the hepatic master regulator hepcidin (HAMP) [11]. In response to iron repletion, hepcidin binds to its receptor, FPN, and causes it to internalize and degrade. This interaction, consequently, blocks the efflux of iron from the enterocytic cell [12].

Iron bioavailability from microalgae was investigated in a few published studies [13,14,15,16]. All of these studies have tested full-fat microalgae as an iron source. The potential of defatted microalgae as a dietary source of iron has been reported previously by our group [17]. In that study, we demonstrated the effectiveness of defatted *Nannochloropsis oceanica* (DGM) in improving growth performance and blood hemoglobin (Hb) and hematocrit status in anemic pigs. Notably, the observed benefits were comparable between DGM and ferrous sulfate, suggesting that when matched for total iron intake, DGM functioned at least equivalently to ferrous sulfate as an iron source. Despite its desirable nutritional composition, microalgae has found limited use in food applications. Even at low inclusion rates, microalgae can adversely impact the sensory and textural profile of foods [18,19,20], due to its powder-like consistency, strong color and fishy smell [21]. Therefore, we hypothesized that using iron extracted from DGM (Fe-DGM) might help to overcome these limitations, and in turn, enable its use in food products. Since blood Hb concentrations may remain unaffected by dietary iron supplements in animals under marginal or moderate iron deficiency [22], a more sensitive biochemical testing may be needed to determine the effects of Fe-DGM on the body iron status of animals under such conditions. The potential of Fe-DGM as a dietary iron supplement has not been investigated previously.

One potential cause of poor iron bioavailability in many plant-based diets is due to high concentrations of phytate. Negative effects of phytate on iron and other element bioavailability can be mitigated by adding phytases to feed or food [23,24]. Inclusion of 500 units of microbial phytase per kilogram of feed improved intrinsic iron bioavailability and absorption, resulting in heavier animals with normal Hb and hematocrit values [17]. Because studies to date have focused on microbial phytase, the similar potential of native plant phytases has been understudied. Our laboratory has also shown the effectiveness of 4% inulin supplementation in improving bioavailability of iron from corn and soybean meal diets to anemic young pigs [25]. These effects were associated with upregulated expression of intestinal iron transporters DMT1, DCytB and FPN [26,27]. Such beneficial effects can be attributed to a few reasons. Inulin is a substrate for gut bacteria that synthesize short chain fatty acids (SCFA). SCFA can increase iron solubility in the lumen, making iron more readily available for absorption [25]. Additionally, inulin can also stimulate enhanced mucin secretion. Since DMT1 may also be secreted alongside mucin, it may offer more opportunities to bind iron and facilitate its uptake [26]. However, our studies have not revealed associations between increased iron uptake and pH or microbial phytases [25]. In contrast, combining 6% inulin and a bacterial phytase at 1000 units/kg diet did not improve growth performance or mineral status in piglets [28]. That study used non-anemic pigs, and the high dose of inulin was impractical for human nutrition. On the other hand, additive or synergistic effects of inclusions of minute amounts of plant phytase and inulin together as promoters of dietary iron utilization by anemic animals were not explored.

Therefore, we performed this experiment to determine the effects of Fe-DGM (33 mg Fe/kg of diet) and a combination of 1% inulin and wheat phytase at 250 U/kg of diet (INU-PHY) on (1) Hb repletion and non-heme iron and ferritin accumulation and (2) iron metabolism-related gene expression in the liver and duodenum of moderately anemic mice fed a phytate-rich diet. Our findings demonstrate that Fe-DGM enhanced blood Hb concentrations and body iron stores, while the INU-PHY diet resulted in modest improvements in dietary iron utilization through a rice-based inhibitory diet.

## 2. Materials and Methods

### 2.1. Extractions and Diets

Soluble iron was extracted from DGM (Cellana, Kailua Kona, HI, USA) using the method of Eagling et al. with minor modifications [29]. Briefly, 2 g DGM was extracted with 10 mL of Tris HCl buffer (50 mM, pH 7.5) at 37 °C for 18 h. Samples were then centrifuged for 10 min at 21,000× *g*. The amount of extracted iron was determined in the supernatant using bathophenanthroline assay, following reduction with L-ascorbic acid (Sigma-Aldrich, St. Louis, MO, USA). Wheat bran and red fife were donated by Maine Grains (Skowhegan, ME, USA), and medina wheat was donated by Dr. M.E. Sorrells from Cornell University. Phytase was extracted from the wheat samples overnight at 4 °C using cold water [30], and the phytase activity was determined as previously described [31]. Following extraction, the Fe extract was oven-dried at 50 °C to 90% dryness (15 h) for incorporation into diets. Due to the small volume of phytase extract used in diet supplementation, the phytase extracts from wheat bran were added as is. Inulin (Raftiline HP, long chain, degree of polymerization = 10–60, average of 25) was provided by Beneo-Orafti (Parsippany, NJ, USA). The polished rice (Asian Best Red Elephant Brand) was purchased from a local market, and contained 6 mg Fe/kg. The rice was powdered using a coffee grinder (Krups F203, Solingen, Germany) before incorporation into diets. The composition of the different diets is presented in Table 1. Other than their iron content, the diets were formulated to meet the nutritional requirements of rodents as recommended by the National Research Council [32].

### 2.2. Animals and Sample Collection

C57BL/6 mice (7 weeks old, *n* = 24) bred at Cornell University’s mouse facility were housed in a controlled environment (12 h daylight cycle, lights off at 18:00) and provided with free access to food and water. The mice were allocated to different dietary treatments (*n* = 8/group; 5 males, 3 females) based on body weight, litter, gender and Hb concentrations. Before the beginning of the experiment, the mice were fed a rice-based basal diet with no supplementary iron (control, 6 mg Fe/kg) for 10 weeks to deplete their body iron stores. The mice were fed continuously either the control diet or control + Fe-DGM (39 mg Fe/kg), or control + INU-PHY (6 mg Fe/kg) for 6 weeks. The body weights of mice were recorded weekly after overnight fasting. Animals were deprived of food for 8 h overnight before blood collection. For Hb determination, blood was drawn via tail bleeding. At the end of the study, the animals were euthanized in carbon dioxide chambers. Tissue samples of liver, muscle, spleen, and duodenum were collected for downstream processing and stored at −80 °C until further analysis. The animal experiment was approved by the Institutional Animal Care and Use Committee at Cornell University (No. 2007-0008).

### 2.3. Dosage Information/Dosage Regimen

Animals had free access to diets containing microalgal iron (33 mg Fe/kg), or supplemental inulin (1%) and plant phytase (250 units/kg). Based on an average daily feed intake of approximately 4 g [33], this dose provides 3.44 mg microalgal iron per kg body weight per day to the mouse. The equivalent human dose is 0.27 mg iron per kg body weight per day (or 16.7 mg iron per day for a 60 kg adult) [34]. An equivalent human dose of inulin translates to 110.5 mg per kg body weight per day (537.5 mg per day for 60 kg adult). Both of these doses are reasonably achievable in humans.

### 2.4. Hematology

Hb was measured using the cyanmethemoglobin assay method as per the manufacturer’s instructions (Pointe Scientific, Canton, MI, USA). Total body hemoglobin iron (Hb-Fe) was calculated as described by Monsen et al. [35].

### 2.5. Tissue Non-Heme Iron Concentration

Briefly, a 100 mg tissue sample was added to 250 µL of 40% trichloroacetic acid in 6 N HCl (1:1 *v/v*), followed by 10 µL of 0.39% NaNO_2_. Non-heme iron was then determined in the tissues as previously described [36].

### 2.6. Quantitative Real-Time PCR

Total mRNA was isolated from duodenum and liver cross-sections using the TRIzol Reagent (Invitrogen, Carlsbad, CA, USA). The RNA integrity was assessed using the Agilent Bioanalyzer 2100 and RNA 6000 Labchip kit (Agilent Technologies, Santa Clara, CA, USA). The key genes involved in iron metabolism—liver (HAMP) and duodenum (DMT1, DCytB, FPN, Tf, TfR1), along with the housekeeping gene (β-actin)—were quantified as described previously [27]. The primer sequences are listed in Table 2.

### 2.7. Western Blotting

After liver samples were homogenized [27], 30 µg of protein was loaded onto 12% SDS-polyacrylamide gels, and probed with a rabbit anti-mouse ferritin antibody (1:1500) (Abcam, Cambridge, MA, USA) and a goat anti-rabbit secondary antibody (1:3000) (Bio-Rad, Hercules, CA, USA). The resultant ferritin protein bands were visualized using SuperSignal West Pico chemiluminescent substrate system (Thermo Fisher Scientific, Waltham, MA, USA).

### 2.8. Phytate Content

Phytate content was determined in milled rice and DGM using a commercially available kit (K-PHYT, Megazyme, Wicklow, Ireland) following the manufacturer’s instructions, and values were expressed in µg/g.

### 2.9. Total Phenolic Content

Total phenolic content in DGM and diet samples were determined in acidified methanolic extracts (methanol and 1M HCl, 85:15 *v/v*) using the Folin–Ciocalteu method [37]. Two biological replicates were run in triplicate for each sample. All values were expressed as gallic acid equivalents (GAE) (mg GAE equivalents/g sample).

### 2.10. Statistical Analysis

Data were analyzed using one-way analysis of variance for the main effect of treatments. Mean differences were compared using Tukey’s post-hoc test. For mRNA expression results, the *t*-test (unpaired, Welch’s correction) was used to compare differences with the control. Treatment differences were considered significant at *p* < 0.05 (Prism, v 7.0, GraphPad Software, La Jolla, CA, USA). Values are expressed as means ± SE.

## 3. Results

### 3.1. Soluble Iron, Phytase, Phytate and Total Phenolic Concentrations

**Figure 1:**
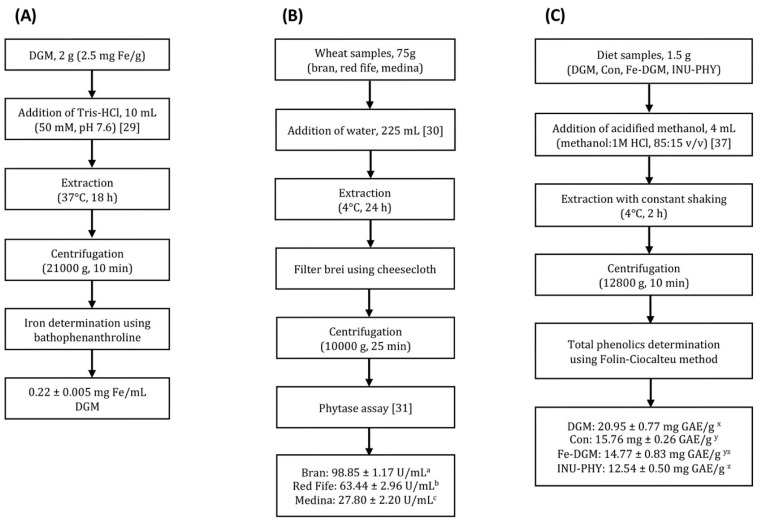
(**A**) Methodology for extracting iron from defatted *Nannochloropsis oceanica* and measurement using phenanthroline assay; (**B**) methodology for extracting phytase from different wheat products and phytase activity determination; (**C**) methodology for extracting total polyphenolic content of *Nannochloropsis oceanica* (DGM) and experimental diets. Data are expressed as means ± SE. Within the same panel, means that do not share the same letter (a, b, c, x, y, z) are significantly different (*p* < 0.05).

Figure 1A shows the procedure of extracting iron from DGM and the final concentration of soluble Fe in the preparation (0.22 mg Fe/mL DGM). Phytase was extracted from three different wheat samples (Figure 1B). Compared to red fife and medina, wheat bran had a higher (*p* < 0.0001) phytase activity (63.44 vs. 27.80 vs. 98.85 U/mL respectively). Therefore, phytase extracted from wheat bran was used for experiments in this study. The concentration of phytate in the polished rice used to prepare the experimental diets was 0.82 µg/g, while DGM had 5.70 µg/g phytate. The measured total phenolic content of DGM and the three diets is shown in Figure 1C. DGM had the highest (*p* < 0.05) phenolic concentration at 20.95 ± 0.77 mg GAE/g, followed by control diet (15.76 ± 0.26), Fe−DGM diet (14.77 ± 0.83) and INU-PHY diet (12.54 ± 0.50). There was a significant difference between the control and INU-PHY diets.

### 3.2. Growth Performance and Hematological Profile of Mice

**Figure 2:**
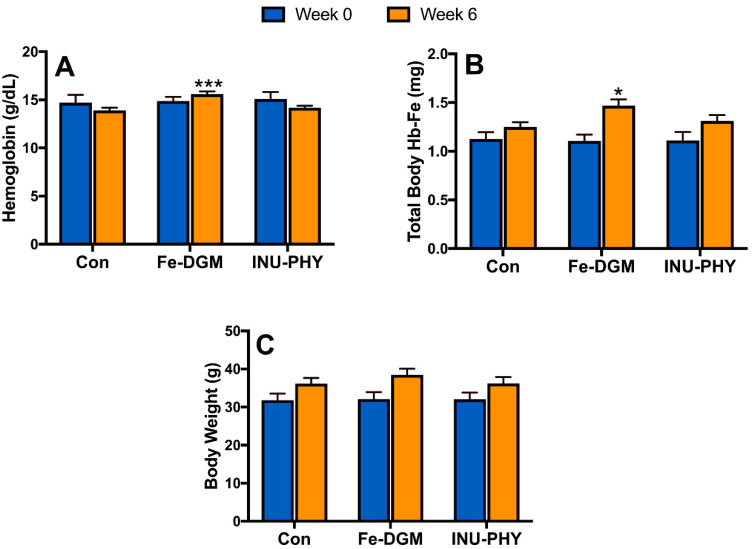
The effect of fortifying iron-deficient rice-based diet (Con) with inulin (1%) and phytase (250 U/kg, INU-PHY), and iron extracted from DGM (33 mg Fe/kg, Fe-DGM) on (**A**) hemoglobin; (**B**) total body Hb-Fe; (**C**) body weight. Data are expressed as means ± SE. Means with a * or *** denote significant difference (*p* < 0.05) or (*p* < 0.001) from the control, respectively; *n* = 7–8.

There were no significant body weight differences between treatments by the end of the study (Figure 2C). Given that the mice had similar Hb levels across treatments at week 0, mice fed the Fe−DGM diet had elevated (*p* < 0.01) blood hemoglobin concentrations (Figure 2A) by week 6 over the other dietary treatments. Similarly, Fe-DGM resulted in greater (*p* < 0.05) total body Hb-Fe (Figure 2B). The blood Hb concentration or total body Hb-Fe of mice were unaffected by addition of inulin and phytase to the control diet.

### 3.3. Body Iron Stores and Relative mRNA Levels of Iron Metabolism-Related Genes of Mice

**Figure 3:**
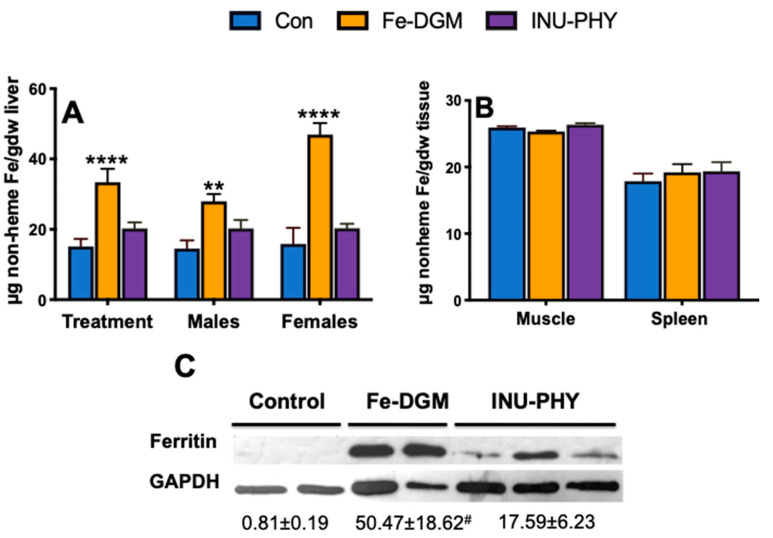
The effect of fortifying iron-deficient rice-based diet (Con) with inulin (1%) and phytase (250 U/kg, INU-PHY), and iron extracted from DGM (33 mg Fe/kg, Fe-DGM) on (**A**) liver non-heme iron; (**B**) muscle and spleen non-heme iron; and (**C**) liver ferritin. Data are expressed as means ± SE. Means with a ** or **** denote significant difference (*p* < 0.01) or (*p* < 0.0001) from the control, respectively; ^#^ *p* = 0.06; *n* = 7–8.

**Figure 4:**
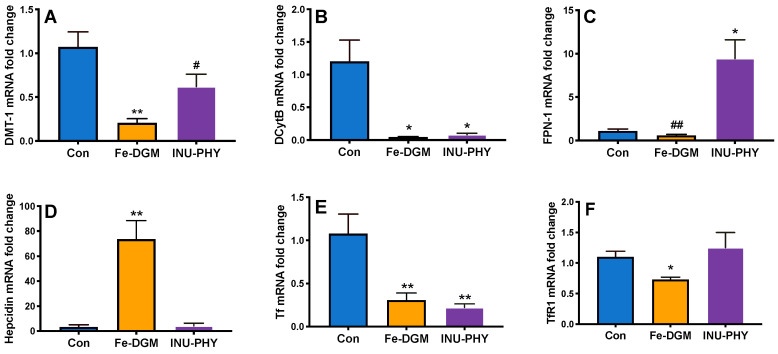
The effect of fortifying iron-deficient rice-based diet (Con) with inulin (1%) and phytase (250 U/kg, INU-PHY), and iron extracted from DGM (33 mg Fe/kg, Fe-DGM) on mRNA levels of (**A**) duodenal DMT1; (**B**) duodenal DCytB; (**C**) duodenal FPN-1; (**D**) liver hepcidin; (**E**) duodenal Tf; (**F**) duodenal TfR1. Data are expressed as means ± SE. Means with a * or ** denote significant difference (*p* < 0.05) or (*p* < 0.01) from the control, respectively; ^#^ *p* = 0.07; ^##^ *p* = 0.08; *n* = 5–6.

As shown in Figure 3A, the Fe-DGM diet enhanced liver non-heme iron by two-fold (*p* < 0.0001) over the control (33.4 ± 3.84 vs. 15.1 ± 2.15 µg/g). Compared with the control, mice fed the inulin and phytase diet had higher non-heme iron amounts in the liver (20.27 ± 1.70 µg/g). However, the difference between the two groups was not significant. There were no treatment differences in the muscle and splenic non-heme iron deposition (Figure 3B). The inclusion of Fe-DGM in mouse diets led to an increase in liver ferritin (*p* = 0.06) over the control (Figure 3C). Compared with the control, the Fe-DGM group had increased (*p* < 0.01) hepatic hepcidin mRNA (23-fold) (Figure 4D), and decreased (all *p* < 0.05) duodenal mRNA levels of DMT1 (19%), DCytB (26-fold), Tf (28%) and TfR1 (34%) (Figure 4A,B,E,F). The mRNA levels of FPN was also reduced by the Fe-DGM diet over control diet; however, this decrease was not significant (*p* = 0.08) (Figure 4C). In comparison with the control, the diet supplemented with inulin and phytase had 8.5-fold greater (all *p* < 0.05) FPN mRNA levels (Figure 4C), whereas mRNA abundances of DCytB and Tf were decreased by 14% and 20%, respectively (Figure 4B,E). Similarly, the mRNA levels of DMT-1 was lower (*p* = 0.07) in INU-PHY mice than control mice.

## 4. Discussion

The major finding from our study was the effectiveness of iron extracted from DGM in improving the iron status in moderately anemic mice, as shown by elevations in blood Hb, total body Hb-Fe, and hepatic ferritin and non-heme iron. While the form of supplemental microalgal iron was novel, the effectiveness was similar to the microalgal biomass [13,14,15,16,38,39]. The improved blood Hb concentrations by the supplemental iron extracted from DGM reaffirm our previous finding that the intrinsic iron from DGM was highly bioavailable [17], and was readily utilized by the mice for Hb production. Dietary inclusions of 1% inulin and 250 U/kg phytase did not enhance blood Hb concentrations over the control. In an earlier study, our laboratory demonstrated that 4% dietary inulin supplementation improved blood Hb concentrations of anemic pigs by up to 28%, while 2% supplementation caused moderate but insignificant Hb increments [25]. Since the growth performance was positively affected by Fe-DGM, there seemed to be no adverse effects at the current fortification rate. Our laboratory is currently verifying the safety of DGM in mice models.

Low iron stores precede anemia development [40]. Thus, an accurate measurement of tissue iron stores can possibly serve as a physiological index for absorbed iron [41]. Indeed, ferritin was severely depleted in control livers. In contrast, supplemental iron extracted from DGM improved (*p* = 0.06) liver ferritin stores of moderately anemic rodents. Remarkably, supplemental inulin and phytase led to 34% higher non-heme liver iron stores over the control. These improved iron utilization responses were likely due to the hydrolyzing action of the wheat bran phytase on the rice phytate, which would enhance the bioavailability of the iron. Such changes could be expected to become significant with longer exposure times. The greatest response (*p* < 0.0001) was seen in the non-heme iron levels in Fe-DGM animals. Most notably, there was a higher (*p* = 0.0035) non-heme iron gain in female livers than that of male livers in the same group. It is possible that such gender-related differences could be due to hormonal mechanisms [42]. However, the mice were young adults at the time of testing, and since there were no gender-related differences in other measures within and across treatments, potential hormonal effects were likely not significant in this case.

Transferrin, the iron transport protein, is mainly synthesized in the liver [43]. However, several studies demonstrate its presence in the intestinal mucosa and epithelial cells, with an enhanced response under iron deficiency [44,45,46,47]. Reduced mRNA levels of DMT1, DCytB, TfR1 and Tf reflect a homeostatic decrease in cellular iron uptake. In the Fe-DGM livers, an increase in hepcidin mRNA levels was consistent with elevations in non-heme iron, suggesting that a positive relationship between hepcidin and the amount of bioavailable iron exists. These iron-dependent changes exhibit an improved iron status of mice fed supplemental microalgal iron. From a physiological perspective, reduced DCytB mRNA, as observed in the inulin and phytase group, may lower the capability to reduce iron to its ferrous form. However, unlike humans, mice can endogenously produce ascorbate [48]. This biosynthetic ability could have played a role in providing reduced Fe for cellular uptake. Regardless, the high FPN mRNA levels in the inulin-phytase mice would increase the rate of iron efflux to the blood. A potential mechanism to explain the well-repeated benefit of inulin in upregulating iron transporter genes [26,27] could be its effect on increasing intestinal villus height and deeper crypts, thereby increasing nutrient absorptive surface area [49]. Consistent with this finding, the inulin and phytase group had greater liver iron stores than the control, although this difference was not significant. However, it remains unclear why FPN was downregulated in moderately anemic control mice. As DMT1, FPN, and DCytB mRNA responses under Fe-deficiency show a strong correlation to corresponding changes in protein levels [48,50], we did not determine protein amounts in this study. The consistent effects of supplemental Fe-DGM, and inulin and phytase, on Hb repletion, ferritin and non-heme iron accumulation agrees with the above responses.

While we recognize that our DGM extract did not include just Fe, we anticipate that compounds of biological importance were probably removed during the extraction process. A detailed discussion is provided in supplementary Document S1. In comparison with the control, the addition of inulin decreased (*p* < 0.05) the overall total phenolic content of the inulin and phytase diet. This could be attributed to the ability of inulin to bind with phenolic compounds, thereby partially masking its measurement in the assay [51].

One of the caveats of this study is the use of young adult mice with moderate growth. The standard rodent chow diet has an estimated nine times more iron (~350 mg Fe/kg diet) than their daily requirements [52]. As a result, older mice develop large iron stores [53] that may make them somewhat resistant to iron depletion. Therefore, researchers often use phlebotomy or phenylhydrazine hydrochloride to induce anemia [54,55,56,57]. Since the etiology of human iron deficiency is largely diet-based, the beneficial effects observed in this study could possibly be of greater extent in anemic adult humans.

In summary, our results demonstrate for the first time that Fe supplementation through a microalgae iron extract (33 mg Fe/kg diet) improved body iron stores and blood Hb concentrations in moderately anemic mice. An improvement in Fe status was also evidenced through favorable responses from iron pathway genes. Microalgae are being promoted for biofuel, protein and other bioactive compound production, and as such, algal farming is highly subsidized by the governments of some emerging countries (such as India). As a result, India has become one of the largest producers of Spirulina [58]. The inclusion of an additional iron extraction step may affect the feasibility of DGM in commercial applications, but it may also help overcome some of the sensory challenges associated with microalgae use in foods. A combination of minute amounts of inulin and wheat bran phytase modestly enhanced the utilization of iron present in rice, and may benefit over half the world’s population that consider rice as a staple. With over 1.2 billion people affected by iron deficiency anemia, there is an urgent global need for effective iron fortificants that can be successfully used in food products. Accumulating evidence suggests that universally supplementing iron to infants in malarial-endemic areas can result in adverse outcomes. These include increasing intestinal inflammation and enhancing the abundance of enteric pathogens in Côte d’Ivoire [6] and Kenya [7], elevating the incidence of bloody diarrhea in Pakistan [59], and a higher morbidity and mortality risk in Zanzibar [8]. Therefore, it is ideal to minimize the total iron content of diets, but ensuring sufficient bioavailable iron through the promotion of such iron enhancers. In diets where staple foods predominate, iron fortification could be successful, and potentially safer, if a food-based solution containing low amounts of DGM as a source of highly bioavailable iron, with bran phytase and inulin as agents maximizing iron release, is implemented.

## Figures and Tables

**Table 1:** Composition of the experimental diets (in %)

Diet	Control	Fe-DGM	INU-PHY
Rice ^a^	41.70	41.70	40.70
Sucrose	30.00	30.00	30.00
Casein	10.45	10.45	10.45
Corn Oil	8.00	8.00	8.00
Gelatin	5.00	5.00	5.00
Calcium Carbonate	1.20	1.20	1.20
Vit/Min premix ^b^	2.00	2.00	2.00
Fe-DGM mixed with rice ^c^	-	1.00	-
Methionine	0.30	0.30	0.30
Choline	0.20	0.20	0.20
Selenium	0.15	0.15	0.15
Inulin	-	-	1.00
Phytase mixed with rice ^d^	-	-	1.00
Calculated values	-	-	-
Crude Protein, %	16.60	16.00	16.00
Crude Fat, %	7.90	7.20	7.80
Fe, mg/kg	6.00	39.00	6.00

^a^ Fe: 6 mg/kg; ^b^ provided per kg of diet: Zn, 10 mg as ZnCl_2_; Cu, 6 mg as CuSO_4_; Mn, 10 mg as MnSO_4_; I, 0.15 mg as KI; P, 3 g as KH_2_PO_4_; K, 2 g as KCl; Na, 500 mg as NaCl; Mg, 500 mg as MgSO_4_; thiamine, 5 mg; riboflavin, 7 mg; vitamin B_6_, 8 mg; Ca panthothenate, 16 mg; niacin, 15 mg; vitamin K, 1 mg; folic acid, 0.5 mg; vitamin E, 22 IU; biotin, 0.2 mg; vitamin B_12_, 0.01 mg; vitamin A, 2400 IU; vitamin D, 1000 IU.; ^c^ iron extracted from defatted *Nannochloropsis oceanica* provided 33 mg Fe per kg of diet.; ^d^ wheat bran-extracted phytase provided 250 units of activity per kg of diet.

**Table 2:** List of primers used for qPCR analysis.

Gene ^a^	Forward (5′ to 3′)	Accession Number
	Reverse (3′ to 5′)	
β-actin	CACCCTGTGCTGCTCACC	NM_007393
	GCACGATTTCCCTCTCAG	
DCytB	CATCCTCGCCATCATCTC	AF354666
	GGCATTGCCTCCATTTAGCTG	
DMT1	GGCTTTCTTATGAGCATTGCCTA	L33415
	GGAGCACCCAGAGCAGCTTA	
FPN	TTGCAGGAGTCATTGCTGCTA	AF226613
	TGGAGTTCTGCACACCATTGAT	
TfR1	TCATGAGGGAAATCAATGATCGTA	X57349
	GCCCCAGAAGATATGTCGGAA	
HAMP	CCTATCTCCATCAACAGAT	AF297664
	TGCAACAGATACCACACTG	
Tf	ATACCGATGCTATGACCTTGGAT	NM_133977
	CAGGACTTCTTGCCTTCGAG	

^a^ Abbreviations: DCytB, duodenal cytochrome B; DMT1, divalent metal transporter 1; FPN, ferroportin; TfR1, transferrin receptor 1; HAMP, hepcidin; Tf, transferrin.

## Supplementary Materials

The following are available online at https://www.mdpi.com/2072-6643/12/8/2239/s1, Document S1: Extract characterization.
