# Supplementary material for: Supplemental Microalgal Iron Helps Replete Blood Hemoglobin in Moderately Anemic Mice Fed a Rice-Based Diet
Source: Nutrients. 2020 Jul 27;12(8):2239. doi: 10.3390/nu12082239 (PMC7468699; doi:10.3390/nu12082239)
Supplement: Supplementary file 1 [file nutrients-12-02239-s001.pdf]

Supplemental file 1

Extract characterization

The buffer used for extraction (Tris HCl, 50 mM, pH 7.6) would promote protein precipitation [1]. Since the microalgae used in this study was defatted, majority of the lipid or lipid-soluble compounds were likely extracted during biofuel production. Any remaining lipid-soluble compounds, along with the precipitated protein, would pellet during centrifugation, and would not be present in the extract. As our inclusion rate was very low (30 g DGM biomass extract provided 33 mg Fe per kg of mouse feed, 1.4% crude fiber), fiber content in the extract (present if any) should have been negligible. In our previous study [2], we demonstrated that addition of 10% DGM to diets of broiler chickens did not affect glutathione concentration or superoxide dismutase activity in the liver and breast tissues compared with the control. Therefore, the low inclusion of actual microalgae biomass residue did not add sufficient antioxidants or could confound the result in this study. Further research is warranted to fully characterize the presence of any iron promoters in the extract. Because of the high bioavailability of our Fe-DGM extract, it can be concluded that the extract constituents did not interfere with iron absorption.

References

1. Bondos, S.E.; Bicknell, A. Detection and prevention of protein aggregation before, during, and after purification. Anal. Biochem. 2003, 316, 223–231.
2. Tao, L.; Sun, T.; Magnuson, A.D.; Qamar, T.R.; Lei, X.G. Defatted Microalgae-Mediated Enrichment of n–3 Polyunsaturated Fatty Acids in Chicken Muscle Is Not Affected by Dietary Selenium, Vitamin E, or Corn Oil. J Nutr 2018, 148, 1547–1555.
